# Distinct cytotoxic cell subsets underlie protective and non-protective immunity to African swine fever virus

**DOI:** 10.1080/22221751.2026.2685920

**Published:** 2026-06-05

**Authors:** David Marín-Moraleda, Aida Tort-Miró, Enrique Ezcurra, Sergio Montaner-Tarbes, Jordana Muñoz-Basagoiti, Elena Coatu, María Jesús Navas, Marta Muñoz, Paula Monleon, Judith González-Oliver, Lola Pailler-García, Sonia Pina-Pedrero, Francesc Accensi, Fernando Rodríguez, Jordi Argilaguet

**Affiliations:** a Unitat Mixta d'Investigació IRTA-UAB en Sanitat Animal, Centre de Recerca en Sanitat Animal (CReSA), Campus de la Universitat Autònoma de Barcelona (UAB), Bellaterra, Spain; b Institut de Recerca i Tecnologia Agroalimentàries (IRTA), Programa de Sanitat Animal, Centre de Recerca en Sanitat Animal (CReSA), Campus de la Universitat Autònoma de Barcelona (UAB), Bellaterra, Spain; c WOAH Collaborating Centre for the Research and Control of Emerging and Re-Emerging Swine Diseases in Europe (IRTA-CReSA), Bellaterra, Spain; d Departament de Sanitat i d'Anatomia Animals, Facultat de Veterinària, Campus de la Universitat Autònoma de Barcelona (UAB), Bellaterra, Spain

**Keywords:** African swine fever, ASF immunity, cytotoxic cells, live attenuated vaccine, correlates of protection

## Abstract

Limited understanding of African swine fever (ASF) immunity remains a major barrier to the rational development of safe and effective vaccines. While antibody-mediated protection is still poorly defined, growing evidence highlights a central role for cellular immunity, particularly cytotoxic cells, as components associated with ASF virus (ASFV) infection control. However, the contribution of individual cytotoxic subsets across different virological and immunological contexts is not well characterized. Here, we investigated cytotoxic responses during BA71ΔCD2 live attenuated vaccine-induced protection and during late-stage lethal ASFV infection. Protective immunity was characterized by antigen-specific cytotoxic T-cell responses, whereas acute ASF was associated with broad cytotoxic activation. Early increases in perforin-positive CD4^-^CD8αβ^+^ T cells coincided with the onset of protection, and elevated recall responses correlated with survival following lethal challenge, supporting their association with protective immunity. Additional correlates of protection included CD4^+^CD8αβ^+^ cytotoxic T cells, IFNγ-producing cells, and specific antibodies, illustrating the multifactorial nature of protective immunity. In contrast, pigs with acute ASF showed broad cytotoxic expansion, perforin-positive natural killer and γδ T cells showing the strongest association with viremia. Together, these findings advance our understanding of cytotoxic responses to ASFV and identify perforin-positive T cells, alongside complementary immune components, as potential correlates of protection for future vaccine development.

## Introduction

The complexity of immune responses to African swine fever (ASF) remains a major obstacle to developing safe and effective vaccines. ASF virus (ASFV) continues to infect domestic pigs and wild boars worldwide, causing substantial economic losses [1]. Live attenuated vaccines (LAVs) induce strong protection [2], and three were approved in Vietnam [3]. However, biosafety concerns limit their wider deployment [4,5], highlighting the need for safer subunit vaccines. Although progress has been made in identifying protective antigens and delivery platforms [6], vaccine development is hampered by the genetic complexity of ASFV and by an incomplete understanding of the immune mechanisms underlying protection and disease.

Acute ASF pathogenesis is driven by the ability of ASFV to modulate host immune responses, killing infected monocytes and macrophages while interfering with multiple innate and cell-death pathways [7–10]. As a result, cells fail to mount an antiviral state or to develop an effective protective immunity. Additionally, infection induces severe leukopenia as a result of dysregulated inflammatory cytokine responses, leading to cell death affecting both infected and bystander cells [11–13], ultimately leading to rapid immunosuppression and a fatal outcome. This pathological profile contrasts with the non-aggressive, self-limiting infections induced by LAVs or naturally attenuated strains. While mechanisms of attenuation are diverse [2], these attenuated viruses replicate with limited immune disruption, allowing the development of effective adaptive responses, thereby representing valuable models to study protective immune mechanisms against ASFV.

Host immunity to ASFV is highly context-dependent. Antibodies play a relevant but limited role in protection [14], and cellular immunity is necessary for infection control [15]. This complexity is further aggravated by the fact that cross-protection between ASFV genotypes is often incomplete, highlighting the need to identify broadly protective immune mechanisms. Among cellular responses, cytotoxic lymphocytes have emerged as central components of protective immunity. Depletion studies have demonstrated a critical requirement for CD8α^+^ cells in protection [16], a marker expressed by most cytotoxic subsets [17]. However, cytotoxic responses to ASFV can arise in both protective and non-protective settings. Antigen-specific cytotoxic T cells are induced following immunization with attenuated strains [15,18]. In some contexts, cytotoxic γδ T and natural killer (NK) cells are also activated [16,18–20], highlighting the multifaceted nature of cytotoxic immunity associated with protection. However, cytotoxic responses are not restricted to protective contexts. During acute ASF, virulent strains induce early suppression followed by late cytotoxic activation, which might reflect uncontrolled inflammation or disease progression rather than protection [15,21–24]. How individual cytotoxic subsets contribute across these contrasting contexts remains poorly defined.

In this study, we investigated cytotoxic immune responses during LAV-induced protection and acute lethal ASFV infection. Using the cross-protective BA71ΔCD2 LAV prototype [18], we show that CD4^-^CD8αβ^+^ T cells are strongly associated with protection. Early increases in circulating CD4^-^CD8αβ^+^ T cells coincided with the onset of protection after vaccination, and recall responses revealed multifactorial immune correlates involving cytotoxic T cells, IFNγ-producing cells, and antibodies. In contrast, acute ASF was characterized by broad cytotoxic activation, with perforin-positive NK-like cells and CD8αα^+^ γδ T cells showing the highest association with viremia. Overall, this study provides new insights into the context-dependent role of cytotoxic lymphocytes in ASF immunity and helps define immune correlates relevant for rational ASF vaccine design.

## Materials and methods

### Ethics statement

All samples used in this study were derived from animal experimental procedures approved by the Ethics Committee on Animal Experimentation of the *Generalitat de Catalunya* (codes 12433, 12393, 12648 and 11947). All experiments were conducted in BSL-3 facilities at the IRTA-CReSA.

### Samples from animal infections

Samples were obtained from several animal experiments, including BA71ΔCD2-vaccinated pigs described in previously published studies [23,25], as well as unvaccinated pigs undergoing acute ASF after infection with the Georgia 2007/1 strain. A detailed summary of the origin, experimental design, sample type, and figures associated with each dataset is provided in Supplementary Table S1. Further details are included in Supplementary material.

### Viruses

BA71ΔCD2 and Georgia 2007/1 ASFV strains were titrated by immunoperoxidase monolayer assay (IPMA) [26] or hemadsorption assay [27], respectively. Titres were expressed as 50% tissue culture infectious dose (TCID_50_)/mL and 50% haemagglutinating units (HAU_50_)/mL, according to the Reed–Muench method [28]. Further details are included in Supplementary material.

### Flow cytometry

For recall responses, cells from vaccinated pigs were stimulated with either BA71ΔCD2 or Georgia 2007/1. *Ex vivo* analyses of PBMCs from naïve or acutely infected pigs were performed without previous stimulation. Further details are provided in Supplementary material.

### Microfluidic quantitative PCR assay

The microfluidic qPCR protocol is described in Bosch-Camós et al. [18] and detailed in Supplementary material. Data was analysed using the Fluidigm Real-Time PCR Analysis software v4.1.3 and the DAG Expression software v1.0.5.6 [29]. Further details are included in Supplementary material.

### Statistical analyses

Statistical analyses were performed using GraphPad Prism version 10.6.1 (GraphPad Software). The specific statistical tests applied to each dataset are indicated in the corresponding figure legends. Correlation analyses were performed using Pearson’s or Spearman’s tests as appropriate, based on data distribution. Where multiple comparisons were involved, *p*-values were adjusted using the Benjamini–Hochberg false discovery rate (FDR) method. Further details are included in Supplementary material.

## Results

### Early increase of cytotoxic CD4^-^CD8αβ^+^ T cells in BA71ΔCD2-vaccinated pigs correlates with protection

**Figure 1.**
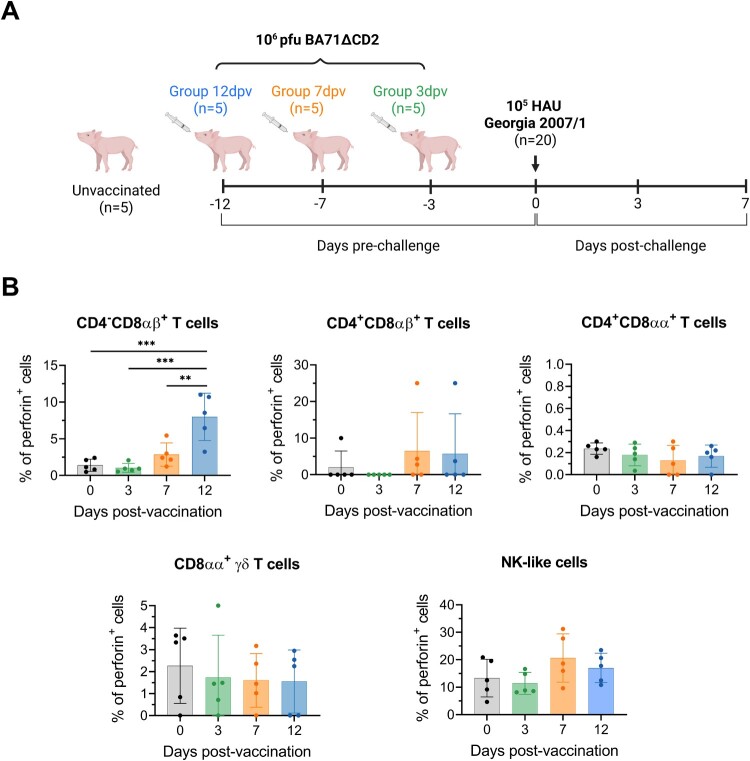
**Expansion of blood cytotoxic CD4^-^CD8αβ^+^ T cells 12 days after BA71ΔCD2 vaccination. (A)** Schematic representation of the experimental design from Marín-Moraleda et al. [23]. (**B**) Percentages of perforin-positive blood cells (within the parental populations) in four experimental groups: unvaccinated pigs (day 0; black), and vaccinated animals challenged at day 3 (group 3 dpv; green), 7 (group 7 dpv; orange) or 12 (group 12 dpv; blue) post-vaccination [23]. Analyses were performed on PBMCs without *in vitro* stimulation by flow cytometry. Statistical significance was determined by one-way ANOVA followed by Tukey’s multiple comparisons test (** *P* ≤ 0.01; *** *P* ≤ 0.001).

To assess the contribution of cytotoxic lymphocytes to the development of protective immunity against ASF, we used the live attenuated vaccine (LAV) prototype BA71ΔCD2 as a model [18]. We first analysed cryopreserved samples from a previously published study that characterized the onset of protective immunity induced by intranasal vaccination with an optimal dose (10^6^ pfu/pig) of BA71ΔCD2 [23]. In that study, pigs were challenged intranasally at 3, 7 or 12 days post-vaccination (3 dpv, 7 dpv and 12 dpv groups), while unvaccinated animals served as controls (Figure 1(A)). As reported, pigs challenged at 3 or 7 dpv showed only a slight delay in disease progression, whereas animals challenged at 12 dpv were able to control infection [23]. Importantly, blood transcriptomic profiling revealed that the onset of protective immunity at 12 dpv was associated with a strong cytotoxic signature [23]. Here, we aimed to determine which cytotoxic cell subsets are associated with this early BA71ΔCD2-induced protection. Frozen peripheral blood mononuclear cells (PBMCs) were collected immediately before challenge (0 days post-challenge (dpc)), and analysed by flow cytometry to quantify perforin-positive CD4^-^CD8αβ^+^ T cells, CD4 ^+^ CD8αβ^+^ T cells, CD4 ^+^ CD8αα^+^ T cells, CD8αα^+^γδ T cells and NK-like (CD3^-^CD8αα^+^) cells (Figure 1(A) and Figure S1). Only CD4^-^CD8αβ^+^ T cells were significantly increased in the protected 12 dpv group (Figure 1(B)). All other cytotoxic subsets showed similar levels across groups, indicating no association with vaccine-mediated protection.

**Figure 2.**
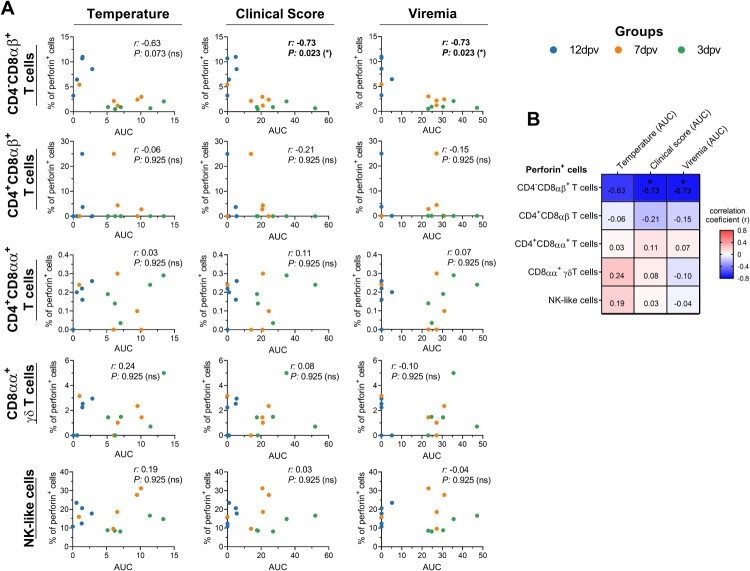
**BA71ΔCD2-induced CD4^-^CD8αβ^+^ cytotoxic T cells in blood correlate with disease control**. (**A)** Correlation between frequencies of perforin-positive cells before challenge and normalized AUC values of rectal temperature, clinical score or viremia after challenge. Dot colours represent vaccinated groups: animals challenged at day 3 (group 3 dpv; green), 7 (group 7 dpv; orange) or 12 (group 12 dpv; blue) post-vaccination [23]. (**B)** Heatmap showing correlation coefficients (r) and significance values for all relationships between ASFV-specific immune parameters and ASF disease control. Correlations were assessed using Pearson’s or Spearman’s as appropriate, according to the criteria described in the Statistical Analysis section (*P* > 0.05 ns; * *P* ≤ 0.05). *p*-values were adjusted for multiple testing using the Benjamini–Hochberg FDR method. Statistically significance refers to FDR-adjusted *p*-values. Significant correlations are highlighted in bold.

We next performed correlation analyses between cytotoxic cell frequencies and protection. For each pig from the three vaccinated groups, we calculated the area under the curve (AUC) for rectal temperatures, clinical scores and viremia measured at different days post-challenge (Figure S2) and compared these values with pre-challenge cytotoxic cell frequencies (Figure 1(B)). Strikingly, only perforin-positive CD4^-^CD8αβ^+^ T cells correlated with milder clinical scores and lower viremia, and a trend to reduced fever (Figure 2). The other cytotoxic subsets did not correlate with any clinical parameters.

### BA71ΔCD2-induced memory cytotoxic CD8αβ^+^ T cells are strongly associated with multifactorial protective immunity

**Figure 3.**
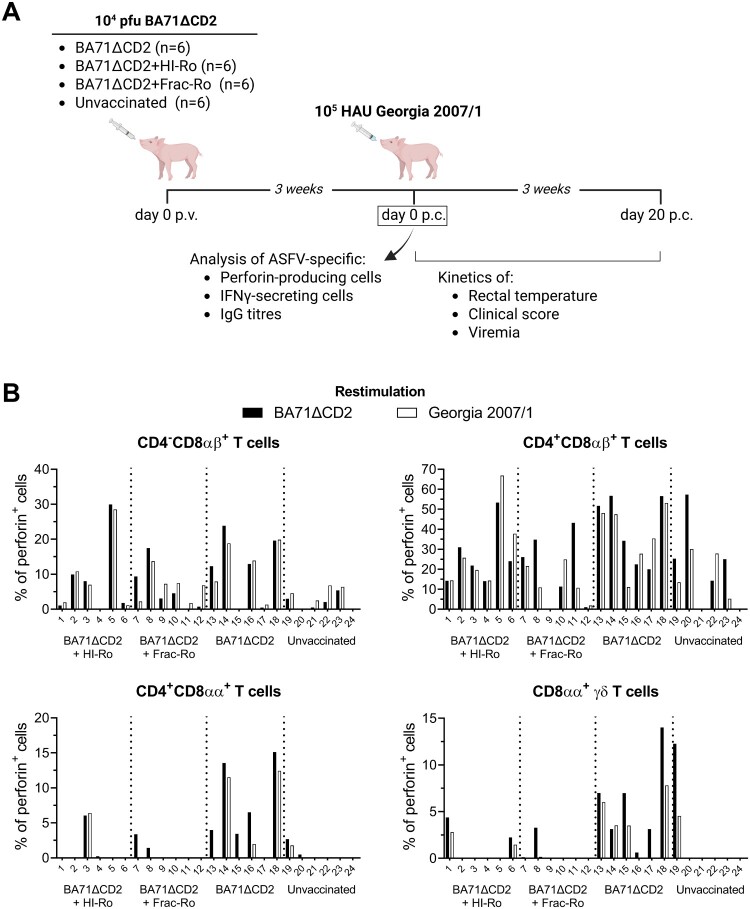
**ASFV-specific cytotoxic T-cell responses in PBMCs from pigs vaccinated with BA71ΔCD2 alone or in combination with the adjuvant prototypes HI-Ro or Frac-Ro.** (**A)** Schematic representation of the experimental design from Tort-Miró et al. [25], indicating the *in vivo* challenge with Georgia 2007/1. (**B)** Percentages of perforin-positive cell subsets within their respective parental populations in PBMCs collected at day 0 post-challenge, following *in vitro* stimulation with BA71ΔCD2 (black bars) or Georgia 2007/1 (white bars), measured by flow cytometry. Background levels from unstimulated cells were subtracted. Numbers on the x-axis correspond to individual pigs.

In addition to cytotoxic T cells, BA71ΔCD2 vaccination induces virus-specific antibodies and polyfunctional Th1 memory responses, including IFNγ-secretion that rapidly activates innate antiviral immunity during recall responses [18]. We therefore sought to determine the relative importance of cytotoxic cells among these vaccine-induced immune components. With this aim, we used samples from pigs vaccinated with a suboptimal intranasal dose of BA71ΔCD2, alone or combined with two adjuvant prototypes (HI-Ro and Frac-Ro) [25] (Figure 3(A)). Instead of boosting BA71ΔCD2-induced immunity, the adjuvant prototypes impaired vaccine efficacy. While 4 out of 6 BA71ΔCD2-vaccinated pigs survived the challenge, only 2 out of 6 survived in each of the adjuvanted groups. However, the survival at the experimental endpoint does not fully reflect disease progression, and this experimental setup resulted in a wide range of outcomes, from acute lethal ASF comparable to unvaccinated controls, to partial protection or complete survival [25]. This heterogeneity enabled identification of correlates of protection.

**Figure 4.**
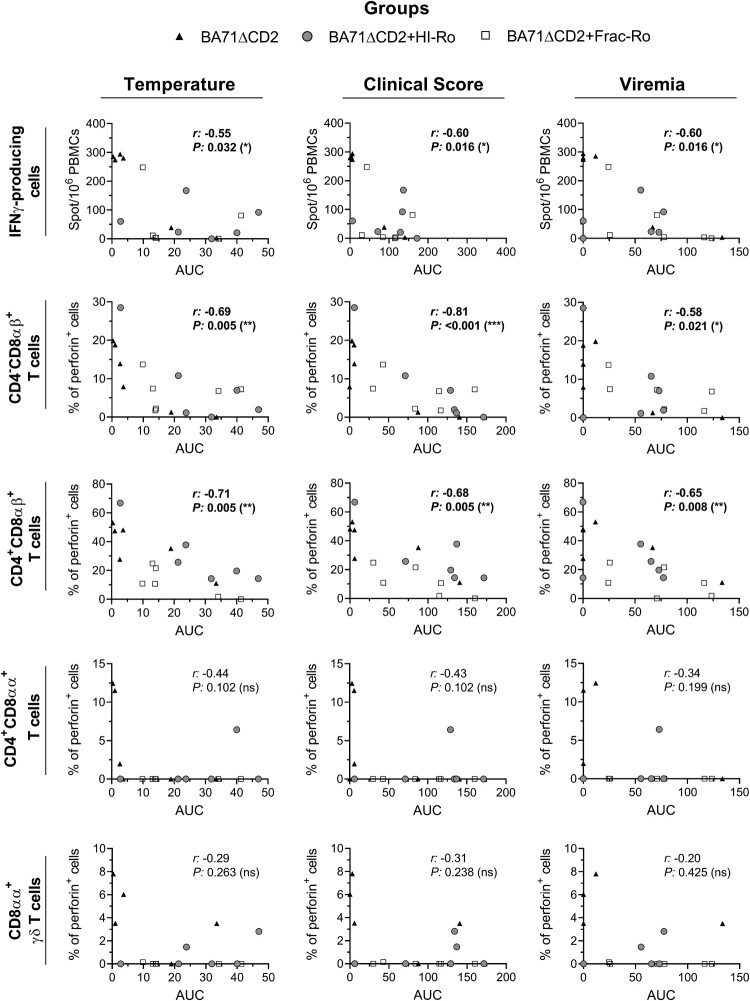
**BA71ΔCD2-induced ASFV-specific cellular responses correlate with protection.** Frequencies of IFNγ-secreting and perforin-positive PBMCs (within the parental populations) before challenge were quantified after stimulation with Georgia 2007/1 and correlated with normalized AUC values of rectal temperature, clinical scores, or viremia. Symbols: black triangles (BA71ΔCD2 alone), grey circles (BA71ΔCD2 + HI-Ro), open squares (BA71ΔCD2 + Frac-Ro). Correlations were assessed using Pearson’s or Spearman’s test as appropriate, according to the criteria described in the Statistical Analysis section (*P* > 0.05 ns; * *P* ≤ 0.05; ** *P* ≤ 0.01; *** *P* ≤ 0.001). *p*-values were adjusted for multiple testing using the Benjamini–Hochberg FDR method. Statistically significance refers to FDR-adjusted *p*-values. Significant correlations are highlighted in bold.

PBMCs collected three weeks after vaccination were stimulated *in vitro* with the virulent Georgia 2007/1 strain to assess recall responses (Figure 3(A)**)**. Cells were also stimulated with the BA71ΔCD2 LAV strain to assess the breadth and consistency of the vaccine-induced memory responses independently of the challenge strain. Perforin-positive cells were quantified by flow cytometry (Figure 3(B)) and IFNγ-secreting cells by ELISpot (Figure S3(A)). Although responses varied among animals, the strongest cytotoxic responses were observed within the CD4 ^+^ CD8αβ^+^ population; however, this subset also showed high background levels in some unvaccinated pigs, indicating low specificity (Figure 3(B)). Correlation analyses using AUCs for rectal temperature, clinical score and viremia (Figure S3(B)) revealed that IFNγ-secreting cells and both CD4^+^ and CD4^-^ CD8αβ^+^ cytotoxic T-cell subsets were significantly associated with protection (Figure 4). In contrast, perforin-positive CD4 ^+^ CD8αα^+^ and CD8αα^+^ γδ T cells did not correlate with any clinical parameter. Consistent with our earlier findings (Figure 2), the strongest correlation was obtained between protection and cytotoxic CD4^-^CD8αβ^+^ T cells when analysed against clinical scores, highlighting their strong association with ASF control (Figure 4). Notably, similar correlation patterns were observed when PBMCs were stimulated with either the vaccine strain or the heterologous virulent strain, supporting the consistency and robustness of the immune parameters identified (Figure S4). To further characterize this cell subset, we assessed the expression of CD45RA and CCR7 to distinguish naïve (CD45RA ^+^ CCR7^+^), central memory T cells (TCM) (CD45RA^-^CCR7^+^), effector memory T cells (TEM) (CD45RA^-^CCR7^-^) and differentiated effector T cells (TDE) (CD45RA ^+^ CCR7^-^) [30]. Protected pigs showed high frequencies of perforin-positive CD4^-^CD8β^+^ T cells after stimulation, distributed between TEM and TDE phenotypes (Figure S5).

**Figure 5.**
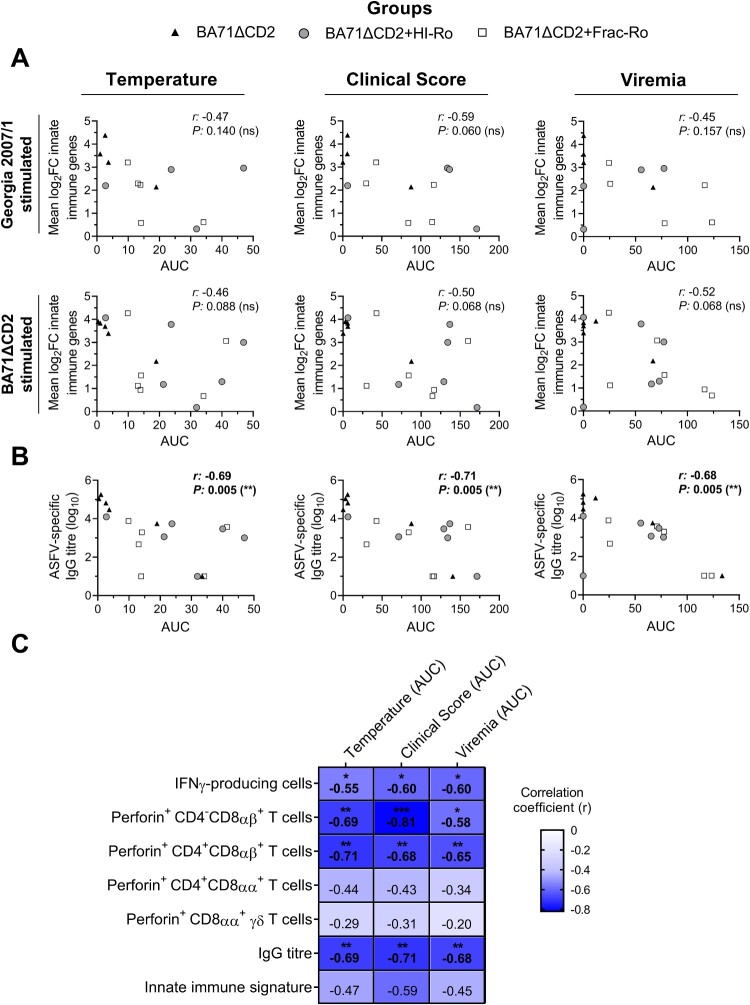
**Multiple immune correlates of protection linked to ASF disease control.** Correlation analyses between normalized AUC values for rectal temperature, clinical scores, or viremia and **(A)** mean of the log₂ fold change (log_2_FC) of innate immune gene expression or **(B)** ASFV-specific IgG titres. Mean log_2_FC values were calculated from gene expression in Georgia 2007/1-stimulated versus unstimulated PBMCs. Symbols: black triangles (BA71ΔCD2 alone), grey circles (BA71ΔCD2 + HI-Ro), open squares (BA71ΔCD2 + Frac-Ro). (**C)** Heatmap summarizing all correlations between ASFV-specific immune variables and disease control. Correlations were assessed using Pearson’s or Spearman’s test as appropriate, according to the criteria described in the Statistical Analysis section (*P* > 0.05 ns; * *P* ≤ 0.05; ** *P* ≤ 0.01; *** *P* ≤ 0.001). *p*-values were adjusted for multiple testing using the Benjamini–Hochberg FDR method. Statistically significance refers to FDR-adjusted *p*-values. Significant correlations are highlighted in bold.

We next contextualized the contribution of cytotoxic CD4^-^CD8αβ^+^ T cells by analysing additional immune components induced by BA71ΔCD2 vaccination. First, we quantified IFNγ-driven innate responses through microfluidic qPCR analysis of 34 innate immune-related genes following stimulation of PBMCs with either Georgia 2007/1 or BA71ΔCD2. Expression profiles varied across animals, with some showing pronounced upregulation and others resembling unvaccinated controls (Figures S6 and S7). The mean log_2_ fold-change across genes served as a proxy for innate recall activation and showed a negative association with protection, although these correlations did not reach statistical significance, regardless of the stimulating virus (Figure 5(A)). Notably, innate activation strongly correlated with IFNγ-secreting PBMCs (Figure S8), supporting the link between inflammatory recall responses and vaccine-induced memory T cells [18]. Finally, ASFV-specific IgG antibodies (Figure S9) correlated significantly with protection (Figure 5(B)), confirming that BA71ΔCD2-induced immunity is multifactorial and includes several potential correlates of protection (Figure 5(C)). Again, similar responses were observed when cells were stimulated with BA71ΔCD2, indicating that the identified immune parameters are robust and consistently detected across related antigenic stimuli (Figures S8 and S10).

### Ongoing acute ASF is associated with broad cytotoxic responses

**Figure 6.**
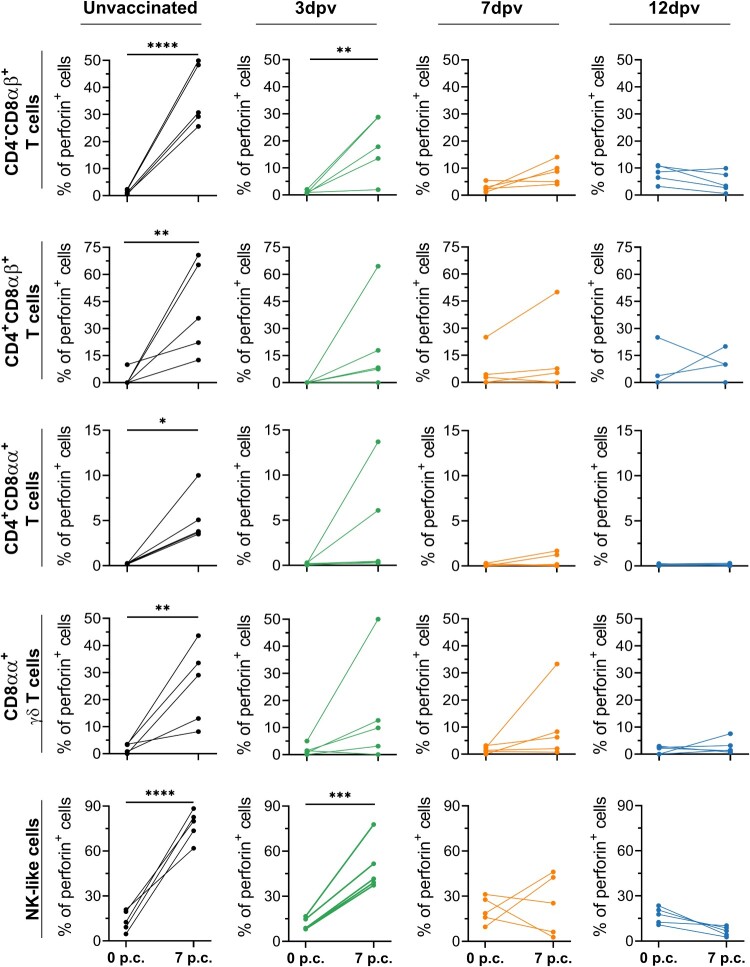
**Broad increase of cytotoxic cells in blood during acute ASF.** Percentages of perforin-positive blood cells (within the parental populations) at days 0 and 7 post-challenge (p.c.) with Georgia 2007/1 from unvaccinated pigs (black), and vaccinated animals challenged at day 3 (green), 7 (orange) or 12 (blue) post-vaccination [23]. Experiments were performed with cryopreserved PBMCs analysed by flow cytometry without prior *in vitro* stimulation. Statistical significance was determined by two-way ANOVA followed by Šidák’s multiple comparisons test (* *P* ≤ 0.05; ** *P* ≤ 0.01; *** *P* ≤ 0.001; **** *P* ≤ 0.0001).

**Figure 7.**
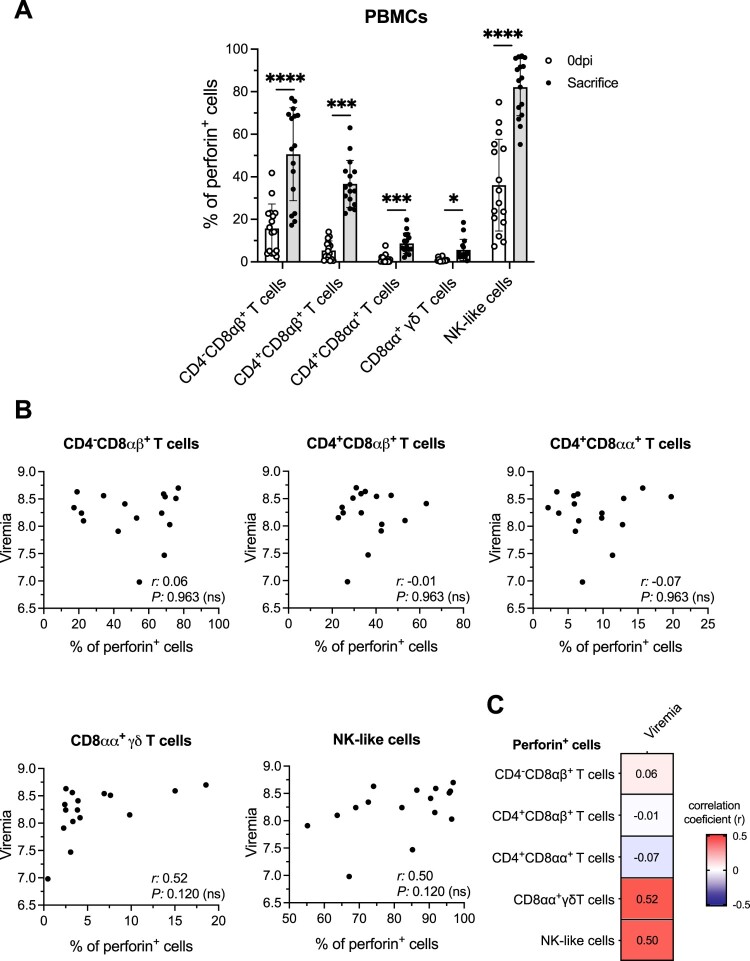
**Perforin-positive NK-like and CD8αα^+^ γδ T cells correlate with viral loads during acute ASF.** (**A**) Frequencies of perforin-positive cytotoxic cell subsets (within the parental populations) measured *ex vivo* in uninfected controls (white bars) and ASFV-infected pigs. Statistical significance was assessed by two-way ANOVA followed by Šidák’s multiple comparisons test. **(B**) Correlations between frequencies of perforin-positive subsets and blood viral loads during late acute disease. Analyses were performed using cryopreserved PBMCs collected from three independent previous studies. Viremia values are represented as Log_10_ gene equivalent copies (GEC)/mL. (**C**) Heatmap summarizing all correlations between frequencies of cytotoxic subsets and viremia. Correlations were assessed using Spearman’s test, according to the criteria described in the Statistical Analysis section (*P* > 0.05 ns; * *P* ≤ 0.05; *** *P* ≤ 0.001; **** *P* ≤ 0.0001). *p*-values were adjusted for multiple testing using the Benjamini–Hochberg FDR method. Statistically significance refers to FDR-adjusted *p*-values.

Cytotoxic activity is also known to increase during acute ASF [15,23,24]. We therefore characterized cytotoxic responses during acute infection and compared them with the protective responses described above. Using cryopreserved PBMCs from the experiment shown in Figure 1(A), we quantified perforin-positive subsets at 0 and 7 dpc with Georgia 2007/1. All cytotoxic subsets were significantly increased at 7 dpc in unvaccinated pigs (Figure 6). In vaccinated animals, the magnitude of this broad cytotoxic response decreased proportionally with the level of vaccine-induced protection, i.e. pigs challenged at 3 or 7 dpv (both unprotected groups) showed moderate cytotoxic responses, whereas fully protected 12 dpv pigs maintained basal levels (Figure 6). To validate these findings in a larger cohort, we analysed cytotoxic subset frequencies in cryopreserved PBMCs from pigs infected either intranasally or by contact with Georgia 2007/1. Animals in late-stage acute disease showed a robust increase in perforin-positive subsets compared with uninfected controls (Figure 7(A)). Correlation analysis showed that the innate cytotoxic lymphocytes NK-like and CD8αα^+^ γδ T cells displayed the highest correlation coefficients with viremia at the time of euthanasia, although not reaching statistical significance (Figure 7(B and C)). In contrast, adaptive CD8αβ T-cell subsets showed no association. Since these analyses were performed using samples from different cohorts (Supplementary Table S1), we evaluated the robustness of these associations by repeating the correlation analyses while iteratively excluding each cohort (leave-cohort-out approach). Similar correlation patterns were observed across all iterations, supporting the robustness of the reported associations despite reduced statistical power (Figure S11).

**Figure 8.**
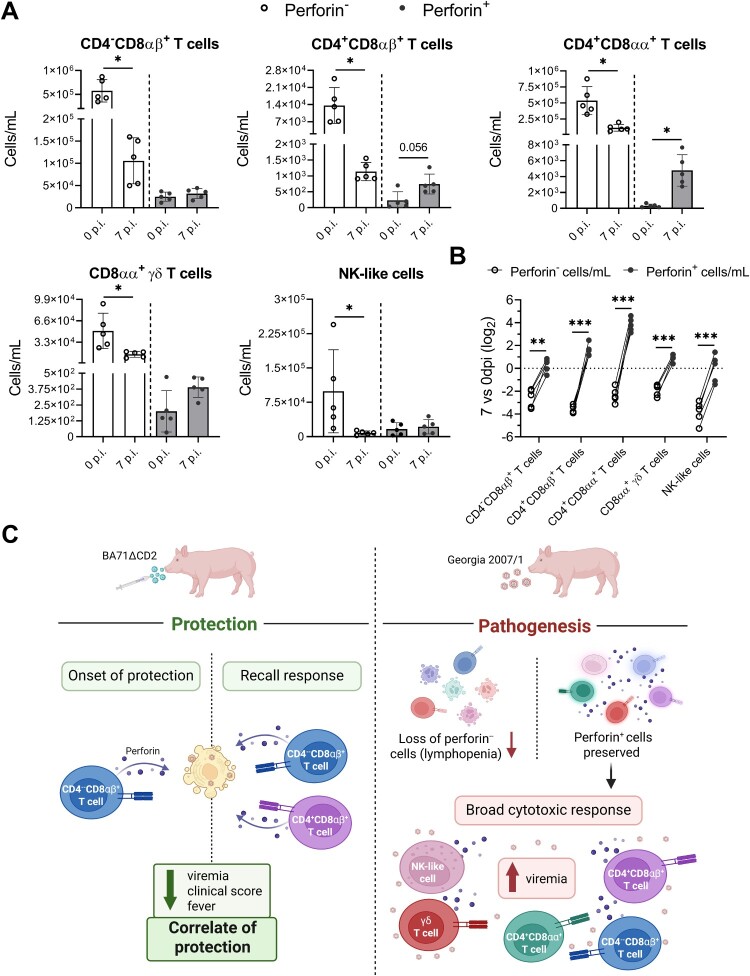
**Perforin-positive cytotoxic cells are less susceptible to lymphopenia during acute ASF.** (**A**) Absolute counts of perforin-positive and perforin-negative populations within the same cytotoxic cell subsets in blood at days 0 and 7 post-infection (p.i.) with Georgia 2007/1. Statistical analysis was performed using multiple Wilcoxon tests with Holm-Šidák correction (* *P* ≤ 0.05). (**B**) Fold-change in these populations at day 7 p.i. relative to pre-infection levels. Cell numbers measured at day 7 p.i. were divided by the corresponding values at day 0 p.i. Statistical significance was assessed by two-way ANOVA followed by Šidák’s multiple comparisons test (** *P* ≤ 0.01; *** *P* ≤ 0.001). (**C**) Graphical abstract comparing cytotoxic immune responses during BA71ΔCD2-induced protection and acute ASF.

Acute ASF is characterized by pronounced lymphopenia during the later stages of infection [31]. To determine whether increases in cytotoxic cell frequencies were simply a consequence of global lymphocyte loss, we infected five pigs with Georgia 2007/1 and quantified both relative frequencies and absolute counts of blood cell populations at 0 and 7 days post-infection. Total T-cell counts confirmed progressive lymphopenia (Figure S12(A and B)). When perforin-positive and -negative populations were analysed separately, the frequency of perforin-positive cells increased after infection, whereas perforin-negative cells either declined or remained stable (Figure S12(C and D)). Absolute counts showed that perforin-positive subsets were largely preserved, and in some cases expanded, unlike their perforin-negative counterparts (Figure 8). Together, these findings indicate that cytotoxic cells exhibit relative resistance to bystander apoptosis, and may undergo active turnover or partial replenishment during acute ASFV infection despite systemic lymphocyte depletion.

## Discussion

Exposure of pigs to virulent or attenuated ASFV strains leads to opposed outcomes; i.e. a rapidly fatal disease or the establishment of protective immunity. Although these trajectories are well characterized, the underlying immune responses remain poorly understood. Here, we show that cytotoxic responses are differentially engaged in these two contexts. During lethal infection with the highly virulent Georgia 2007/1 strain, pigs mounted a broad cytotoxic response involving several lymphocyte subsets. In contrast, the protective cytotoxic activity induced by the LAV BA71ΔCD2 is primarily mediated by CD4^-^CD8αβ^+^ T cells. We also identify ASFV-specific antibodies and IFNγ-producing cells as additional correlates of protection, underscoring the multifactorial nature of ASF immunity. Similar response patterns upon stimulation with genotype I and II strains further suggest that key immune correlates are shared across ASFV genotypes.

Cytotoxic T cells are critical against many viral infections, particularly when neutralizing antibodies provide limited protection, as is the case for ASFV [32]. Although this is especially relevant for non-cytopathic viruses [33], cytotoxic responses also contribute to protection against cytopathic viruses [34,35]. ASFV’s ability to delay apoptosis of infected cells suggests that cytotoxic mechanisms may contribute to restricting early viral replication. Moreover, recent evidence indicates that prompt elimination of infected macrophages may also limit ASFV dissemination through apoptotic bodies [36,37]. Consistent with this idea, vaccine-elicited cytotoxic CD8^+^ T cells might mediate early control of ASFV replication following challenge, as described for other cytopathic viruses [38,39]. The cytotoxic T cell recall responses observed here are in line with findings from other attenuated ASFV strains [15,18,40–43]. However, a detailed characterization of these responses had been lacking. Depletion of CD8α^+^ cells already demonstrated their role in protection [16], but the precise cell subsets involved were not identified. Our results identify cytotoxic CD4 ^+^ CD8αβ^+^ and CD4^-^CD8αβ^+^ T-cell subsets as correlates of vaccine-induced protection. Notably, perforin-positive CD4 ^+^ CD8αβ^+^ cells were also detectable in non-vaccinated animals, indicating that this population is not exclusively vaccine-induced. Furthermore, as perforin detection reflects cytotoxic granule content rather than ongoing degranulation, our analyses describe cytotoxic potential rather than immediate effector activity. These cytotoxic responses are consistent with observations made with other attenuated ASFV strains in previous studies [15,40–42], suggesting that CD8αβ^+^ cells may represent a broader hallmark of effective LAV-induced immunity. As such, this immune component should be targeted by future subunit vaccines aiming to reproduce the protective mechanisms elicited by LAVs. In contrast, CD4^+^CD8αα^+^ T cells did not associate with protection, suggesting a limited contribution of this subset to protective cytotoxic immunity. This is consistent with their association with antigen-experienced or tissue-resident memory phenotypes rather than classical cytotoxic T cells [17,30]. Further functional analyses will be required to clarify their role in the recall response.

Although CD4^-^CD8αβ^+^ T cells were detected in recall responses *in vitro*, their frequencies in blood did not increase early post-challenge in protected pigs. This suggests that control of intranasal ASFV infection may occur locally at mucosal sites, preventing the development of a measurable systemic response. However, this hypothesis remains speculative, as mucosal immune responses were not assessed in this study. Our results are consistent with our intranasal infection model, which mimics more closely the natural exposure than the intramuscular challenge. Considering the recognized importance of tissue-resident T cells in antiviral defence [44], future vaccine efficacy studies should adopt challenge models that better reflect natural ASFV infection dynamics.

In contrast to the protective role of vaccine-induced cytotoxic T cells, in acute ASF a broad cytotoxic activity was observed. While early, vaccine-induced expansion of cytotoxic cells is associated with protection, a late broad cytotoxic activation during uncontrolled viral replication likely reflects dysregulated immunity. Thus, the elevated cytotoxic cell frequencies observed in diseased pigs are likely part of a hyperinflammatory response associated with ASF pathology [45]. The identification of NK and γδ T cells as the subsets showing the highest correlation coefficients with viremia extends previous studies showing elevated perforin-positive subsets in diseased pigs [15,21,22,24,46]. Although these associations might suggest a potential contribution of these innate cytotoxic lymphocytes to antiviral control [47,48], such interpretation would require direct functional validation. Increased cytotoxic activity of these populations, together with αβ T cells, may also reflect enhanced survival, expansion, or resistance to activation-induced cell death, a common feature of acute viral infections [49]. Thus, we cannot exclude that the observed cytotoxic profile is a consequence of the strong inflammatory activation rather than a targeted antiviral response. Moreover, cytotoxic NK and γδ T cells may also promote tissue damage through killing either infected or bystander uninfected cells [50,51]. For a cytopathic virus such as ASFV, direct exacerbation of pathology through macrophage killing by cytotoxic cells is unlikely. However, they might contribute to ASF-associated leukopenia by bystander cell death [24]. Notably, absolute numbers of perforin-positive cells during acute disease were maintained or increased, indicating an active turnover. Thus, increased NK and γδ T cell activity likely reflects a severe inflammatory activation associated with uncontrolled viral replication. However, whether these cells play a functional antiviral role or contribute to immunopathology remains to be determined.

Beyond cytotoxic T cells, our results highlight that BA71ΔCD2-induced protection is multifactorial. This is the first comprehensive analysis aiming to identify multiple correlates of protection induced by a laboratory-attenuated LAV prototype. These correlates derive exclusively from pigs vaccinated with BA71ΔCD2 under diverse experimental conditions. Importantly, in animals receiving the LAV together with HI-Ro or Frac-Ro, the magnitude of vaccine-induced immune responses was reduced due to decreased antigen availability, rather than qualitative alterations of LAV-induced immunity [25]. Nevertheless, comparable multifactorial correlates of protection have been reported in pigs immunized with the naturally, partially attenuated field isolate Estonia 2014, which also induces diverse protective immune responses [52]. These findings support the broader concept that effective antiviral immunity requires coordinated engagement of innate, humoral and cellular compartments [53]. Synergy between vaccine-induced humoral and cellular responses may reduce the immune threshold required for protection [54]. However, additional mechanistic studies are needed to discriminate true causal correlates from surrogate markers of protection in ASF [55]. Antibodies represent another key component of ASFV immunity [14]. We found a strong correlation between ASFV-specific IgG titres and protection, consistent with observations in pigs immunized with the attenuated Pret4Δ9GL strain [56]. ASFV-specific IgG appears concurrently with the onset of BA71ΔCD2-mediated protection [23]. Although antibody-mediated protection remains incompletely understood, neutralizing antibodies induced by ASFV-G-Δ9GL/ΔUK or ASFV-G-ΔI177L LAVs have been associated with protection [57]. However, as these assays were performed with cell-adapted isolates, their physiological relevance remains unclear. The potential role of non-neutralizing antibodies may also contribute to ASFV immunity [58], especially given their importance as correlates of protection in other viral systems [59]. Finally, IFNγ-producing cells emerged among the multiple correlates of protection, and strongly correlated with the inflammatory transcriptional signature observed in stimulated cells. We previously showed that recall responses in BA71ΔCD2-vaccinated pigs involve a positive feedback loop in which IFNγ secreted by Th1 memory cells activates inflammatory macrophages [18]. This rapid induction of innate immunity may restrict ASFV replication in activated macrophages [25,60], contributing to early viral control. Notably, a limitation of this study is that memory responses were assessed only at three weeks post-vaccination, and future work will be required to determine their long-term persistence.

In summary, this study advances the understanding of cytotoxic immunity in ASF by distinguishing protective subsets from those associated with acute disease. CD4^-^CD8αβ^+^ T cells emerge as key correlates of vaccine-induced protection, in contrast to the broad, dysregulated cytotoxic landscape observed during lethal infection. These immune signatures, likely shared by other protective LAV strains, provide a framework for the rational development of safer ASFV vaccines.

## Supplementary Material

Supplementary Table S1_revised.xlsx

Supplementary figures Marin_Moraleda_revised2.docx

Supplementary material Marin_Moraleda_revised2.docx
