# Supplementary material for: Distinct cytotoxic cell subsets underlie protective and non-protective immunity to African swine fever virus
Source: Emerg Microbes Infect. 2026 Jun 5;15(1):2685920. doi: 10.1080/22221751.2026.2685920 (PMC13470925; doi:10.1080/22221751.2026.2685920)
Supplement: Supplementary file 1 [file TEMI_A_2685920_SM4991.docx]

## **Supplementary material**

**Animals**

In all the experiments, six- to eight-week-old domestic pigs underwent a seven-day acclimatation period and had *ad libitum* access to feed and water throughout the experiment. Serum and EDTA-blood samples were collected at the indicated time points. Animal health status was monitored daily according to standardised welfare guidelines. Clinical signs were evaluated assessing behaviour, body condition, presence of cyanosis, and digestive and respiratory signs. Each parameter was scored on a scale from 0 to 3 (0 = normal; 1 = mild; 2 = moderate; 3 = severe). Animals that reached humane endpoints were humanely euthanised.

**Viruses**

BA71ΔCD2 was expanded in COS-1 cells (ATCC). The Georgia 2007/1 ASFV strain was provided by Dr. Linda Dixon (WOAH reference laboratory, Pirbright Institute, UK) and expanded in porcine alveolar macrophages. TCID_50_ values were converted into plaque-forming units (pfu) applying a Poisson distribution (TCID_50_/mL = 0.7 pfu/mL).

**PBMCs from animal infections**

A detailed summary of the origin, experimental design, sample type, and figures associated with each dataset is provided in Supplementary Table S1. Briefly, fresh or cryopreserved peripheral blood mononuclear cells (PBMCs) were obtained from three independent studies: two involving intranasally challenged pigs (10^5^ HAU; n= 11) [[23,25](https://paperpile.com/c/Uwr5XM/V2VW+pAcc)] and one involving pigs (n= 5) infected through an in-contact transmission model [18]. In addition, an *in vivo* experiment was conducted to quantify absolute counts of cytotoxic subsets in fresh PBMCs from intranasally infected pigs (10^5^ HAU; n= 5).

**Cell freezing and thawing**

PBMCs were cryopreserved in foetal bovine serum (FBS) supplemented with 10% dimethyl sulfoxide (DMSO). Once resuspended at the appropriate concentration (10 millions cells/tube), cells were aliquoted into cryovials and gradually cooled to −80 °C, where they were kept overnight. The following day, cryovials were transferred to liquid nitrogen for long‑term storage. The day of use, frozen PBMCs were rapidly thawed in a 37 °C water bath, immediately transferred to pre‑warmed RPMI medium, and washed by centrifugation. After removal of the supernatant, PBMCs were resuspended in fresh RPMI and cells were counted using a Neubauer haemocytometer after staining with Trypan Blue to assess viability. Cell concentrations were adjusted as required before proceeding with downstream assays.

**Quantitative PCR for the detection of ASFV**

Viral genomic DNA was extracted from blood or sera by IndiMag® Pathogen Kit (INDICAL Bioscience) in a semi-automated KingFisher System (Thermo Fisher Scientific). Viral titres were assessed by SYBR Green qPCR targeting the ASFV PK gene [[26](https://paperpile.com/c/Uwr5XM/14Sb)]. Ten-fold serial dilutions of a plasmid encoding the PK gene were used as standard template to quantify viral loads.

**Microfluidic quantitative PCR assay**

PBMCs from BA71ΔCD2-vaccinated pigs were seeded at 10^7^ cells/well in 6-well flat-bottom plates and stimulated overnight with either BA71ΔCD2 or Georgia 2007/1 strains (both at MOI 0.2). Total RNA was extracted using the RNeasy Mini Kit (Qiagen), and the concentration and integrity were measured using the Qubit RNA BR Assay Kit (ThermoFisher Scientific) and the Agilent Bioanalyzer, respectively. cDNA was generated from 160 ng of total RNA using the PrimeScript RT reagent Kit (Takara, Japan). The microfluidic qPCR protocol and the primers used are detailed in Bosch-Camós et al. [18]. Briefly, gene expression was quantified in duplicate using the 96.96 Dynamic Array integrated fluidic circuit of the Biomark HD system (Fluidigm Corporation). Data was analysed using the Fluidigm Real-Time PCR Analysis software v4.1.3 and the DAG Expression software v1.0.5.6 [29]. Relative quantification of target gene expression was performed by normalising to the average expression of three reference genes (YWHAZ, RPL4 and GAPDH) using the 2^−ΔΔCt^ method and expressed as fold change (FC) relative to the unstimulated control samples. Log₂-transformed FC values were used for downstream analyses. Z-scores were calculated from individual log₂FC values for heatmap visualisation, whereas mean log₂FC values per animal were used for correlation analyses.

**Flow cytometry**

For cytotoxic recall responses, cells were stimulated with either BA71ΔCD2 or Georgia 2007/1 at a MOI of 0.2 for 36 hours at 37ºC. RPMI medium and phorbol myristate acetate (PMA, 5 ng/mL) plus ionomycin (500 ng/mL) were included as negative and positive controls, respectively. When indicated, *ex vivo* analyses of PBMCs from naïve or acutely infected pigs were performed without previous stimulation. In all cases, 10^6^ PBMCs were stained for flow cytometry as previously described [[18](https://paperpile.com/c/Uwr5XM/ZS0k)]. PBMCs were stained with LIVE/DEAD Fixable Red Dead Cell Stain Kit (ThermoFisher Scientific). Fc receptors were blocked for 15 min on ice prior to antibody staining. Staining of cells to analyse frequencies of cytotoxic subsets was performed with an antibody mix containing mouse anti-pig CD3ε (PE-Cy7, Clone BB23-8E6-8C8, BD Biosciences), mouse anti-pig CD4α (PerCP-Cy5.5, Clone 74-12-4, BD Biosciences), rat anti-pig γδTCR (APC, Clone MAC320, BD Biosciences), anti-pig CD8α (FITC, Clone 76-2-11, BD Biosciences) and mouse anti-pig CD8β (R-PE, Clone PPT23, Bio-Rad Laboratories). For intracellular staining, cells were treated with the BD Cytofix/Cytoperm Kit (BD Biosciences) and stained with mouse anti-human/pig perforin (eFluor450, Clone dG9, eBioscience). To note, the dG9 monoclonal antibody identifies pre‑existing cytotoxic granule content and therefore reflects cytotoxic potential rather than ongoing degranulation or real‑time effector activity. For immunophenotyping, the anti-CD8α and anti-γδTCR antibodies were replaced with the mouse anti-pig CD45RA (FITC, Clone MIL13, Bio-Rad Laboratories) and the rat anti-human CD197 (CCR7) (APC, Clone 3D12, Invitrogen). For absolute quantification of cell subset numbers, 10 µL CountBright absolute counting beads (Invitrogen) were added to each sample immediately prior to acquisition. Samples were acquired on a BD FACSAria IIu flow cytometer (BD Biosciences), and data was analysed using FlowJo v10.7.1 software (Tree Star Inc).

**Enzyme-linked immunosorbent assay (ELISA)**

ASFV-specific antibodies in pig sera were detected by the WOAH-approved ELISA based on soluble extracts from ASFV-infected cells [18]. Samples were serially diluted from 1/100 to 1/1.562.500 to calculate the endpoint-titration, with a cut-off defined as the average negative control plus three times the standard deviation of the negative control. The secondary peroxidase-conjugate antibody rabbit anti-pig IgG at 1/20000 dilution (Sigma-Aldrich) was used to detect positive sera. Soluble 3,3’,5,5’-tetramethylbenzidine (TMB, Sigma-Aldrich) and H_2_SO_4_ at 1N were used as a specific peroxidase substrate and stop solution, respectively. Plates were read at 450nm and all samples were run in technical duplicates.

**Statistical analyses**

Prior to correlation analyses, data distribution was assessed for normality and homoscedasticity. Pearson’s correlation test was applied when these assumptions were met, whereas Spearman’s rank correlation test was used otherwise. Resulting *P* values were adjusted for multiple comparisons using the false discovery rate (FDR) method. For all other statistical analyses, multiple-comparison corrections are specified in the figure legends. Statistical analyses were performed using GraphPad Prism version 10.6.1 and RStudio version 2023.12.0+369. Graphs were generated using GraphPad Prism and [BioRender.com](http://biorender.com).
