# Supplementary material for: Distinct cytotoxic cell subsets underlie protective and non-protective immunity to African swine fever virus
Source: Emerg Microbes Infect. 2026 Jun 5;15(1):2685920. doi: 10.1080/22221751.2026.2685920 (PMC13470925; doi:10.1080/22221751.2026.2685920)
Supplement: Supplementary file 2 [file TEMI_A_2685920_SM4992.docx]

**Figure S1. Flow cytometry gating strategy. A)** Representative plots used to identify perforin-producing lymphoid cell subsets. **B)** Fluorescence minus one (FMO) controls used to define gates. **C)** Representative unstimulated and virus-stimulated plots.

**Figure S2.** **Kinetics of fever, clinical signs and virus loads after challenge.** Rectal temperatures, clinical scores and viremia from pigs vaccinated 3 (green), 7 (orange) or 12 (blue) days before the challenge. Unvaccinated controls are shown in black. Data were normalised to day 0 post-challenge, and the area under the curve (AUC) was used for correlation analyses. GEC: genomic equivalent copies.

**Figure S3. Normalised rectal temperature, viremia, and clinical scores after challenge.** **A)** Percentages of IFNγ-secreting PBMCs measured by ELISpot at day 0 post-challenge after stimulation with BA71ΔCD2 (black bars) or Georgia 2007/1 (white bars). These results were obtained in a previous study [25], in which the analysis was focused on the group level. **B)** Rectal temperatures, clinical scores, and viremia from pigs vaccinated with BA71ΔCD2 alone or with HI-Ro or Frac-Ro; unvaccinated controls are included. Data were normalised to day 0 post-challenge, and the area under the curve (AUC) values were used for correlation analyses. GEC: genomic equivalent copies.

**Figure S4. Associations between disease control, IFNγ-secreting cells, and cytotoxic cells after BA71ΔCD2 stimulation, showing consistency with results obtained using Georgia 2007/1 stimulation.** Frequencies of IFNγ-secreting and perforin-producing PBMCs (within the parental populations) before challenge were quantified after stimulation with BA71ΔCD2 and correlated with normalised AUC values of rectal temperature, clinical scores, or viremia. Circles: BA71ΔCD2+HI-Ro; squares: BA71ΔCD2+Frac-Ro; triangles: BA71ΔCD2 alone. Correlations were assessed using Pearson’s or Spearman’s test, as appropriate, according to the criteria described in the Statistical Analysis section (*P* > 0.05 ns; * *P* ≤ 0.05; ** *P* ≤ 0.01; *** *P* ≤ 0.001). p-values were adjusted for multiple testing using the Benjamini–Hochberg FDR method. Statistically significance refers to FDR-adjusted p-values. Significant correlations are highlighted in bold.

**Figure S5. Immunophenotyping of BA71ΔCD2-induced cytotoxic CD4^-^CD8β^+^ T cells. A)** Flow cytometry gating strategy used to classify perforin**^+^** CD4^-^CD8β⁺ T cells into naïve (CD45RA⁺ CCR7⁺), central memory (TCM; CD45RA⁻ CCR7⁺), effector memory (TEM; CD45RA⁻ CCR7⁻), and terminally differentiated effector (TDE; CD45RA⁺ CCR7⁻) subsets. **B)** Fluorescence minus one (FMO) controls used to define gates. **C)** Representative unstimulated and virus-stimulated plots. **D)** PBMCs from BA71ΔCD2-vaccinated pigs showing strong protection were stimulated with BA71ΔCD2 or Georgia 2007/1 to assess perforin-producing CD4^-^CD8β⁺ T-cell phenotypes. Statistical analyses were performed using multiple Mann-Whitney tests with Holm-Šidák correction (* *P* ≤ 0.05).

## **Figure S6. Innate immune transcriptomic signatures during the recall response.** PBMCs collected at day 0 post-challenge were stimulated with ASFV Georgia 2007/1. Expression levels of innate immune-related genes were quantified by microfluidic qPCR assay. Heatmap shows z-score normalised log_2_ fold changes (log_2_FC) between stimulated and unstimulated cells. Heatmap columns correspond to individual pigs.

## **Figure S7.** **Innate immune transcriptomic signatures after BA71**Δ**CD2 stimulation.** PBMCs collected at day 0 post-challenge were stimulated with BA71ΔCD2. The heatmap shows z-score normalised log_2_ fold change (log_2_FC) values for innate immune-related genes compared with unstimulated cells.

## **Figure S8.** **IFNγ-secreting cells correlate strongly with innate immune signatures during the recall response**. Correlation analyses between IFNγ-secreting PBMCs (ELISpot) and mean of the log_2_ fold change (log_2_FC) values of innate immune genes after stimulation with Georgia 2007/1. BA71ΔCD2 stimulation was included for comparative purposes, showing consistent patterns of association. Correlations were assessed using Spearman’s test, according to the criteria described in the Statistical Analysis section (*** *P* ≤ 0.001). p-values were adjusted for multiple testing using the Benjamini–Hochberg FDR method. Statistically significance refers to FDR-adjusted p-values.

**Figure S9. ASFV-specific IgG endpoint titres at day 0 post-challenge.** IgG titres from pigs vaccinated with BA71ΔCD2 alone or in combination with HI-Ro or Frac-Ro.

**Figure S10. Associations between ASFV-specific immune responses after BA71ΔCD2 stimulation and disease control.** Heatmap summarising the associations between ASFV-specific immune responses measured upon BA71ΔCD2 stimulation and ASF disease control. These analyses are shown to assess the consistency of immune parameters identified using Georgia 2007/1 stimulation, and not to define correlates of protection. Correlations were assessed using Pearson’s or Spearman’s test as appropriate, according to the criteria described in the Statistical Analysis section (* *P* ≤ 0.05; ** *P* ≤ 0.01; *** *P* ≤ 0.001). p-values were adjusted for multiple testing using the Benjamini–Hochberg FDR method. Statistically significance refers to FDR-adjusted p-values.

**Figure S11. Robustness analysis of correlations between cytotoxic cell subsets and viremia during acute ASF.** Correlation analyses were repeated using a leave-one-cohort-out approach, excluding one cohort at a time (Cohort A, B, or C; specified in Supplementary Table S1). Despite reduced statistical power, similar trends were observed across all analyses, with NK-like cells and CD8αα^+^ γδ T cells consistently showing the strongest positive associations with viremia. Viremia values are represented as Log_10_ gene equivalent copies (GEC)/mL. p-values were adjusted for multiple testing using the Benjamini–Hochberg FDR method. Statistically significance refers to FDR-adjusted p-values.

**Figure S12. Increased frequencies of cytotoxic subsets during acute ASF-associated lymphopenia. A)** Frequencies (within live PBMCs) and absolute counts of the indicated cell populations in blood at 0 and 7 days post-infection (p.i.) with Georgia 2007/1. **B)** Fold-change in these populations at day 7 relative to pre-infection levels. **C)** Frequencies of perforin-positive and perforin-negative populations (within parental subsets) in blood at 0 and 7 days post-infection. **D)** Fold-change of these perforin-defined populations at day 7 relative to pre-infection levels. For B and D panels, cell numbers measured at day 7 p.i. were divided by the corresponding values at day 0 p.i. Statistical analyses for panels A and C were performed using multiple Wilcoxon tests with Holm-Šidák correction (* *P* ≤ 0.05). Significance for panels B and D was assessed by two-way ANOVA followed by Šidák’s multiple comparisons test (** *P* ≤ 0.01; *** *P* ≤ 0.001).
